# Genome-wide recombination rate variation in a recombination map of cotton

**DOI:** 10.1371/journal.pone.0188682

**Published:** 2017-11-27

**Authors:** Chao Shen, Ximei Li, Ruiting Zhang, Zhongxu Lin

**Affiliations:** 1 National Key Laboratory of Crop Genetic Improvement, College of Plant Science & Technology, Huazhong Agricultural University, Wuhan, Hubei, China; 2 College of Agronomy and Plant Protection, Qingdao Agricultural University/Shandong Key Laboratory of Dryland Farming Technology, Qingdao, Shandong, China; Fujian Agriculture and Forestry University, CHINA

## Abstract

Recombination is crucial for genetic evolution, which not only provides new allele combinations but also influences the biological evolution and efficacy of natural selection. However, recombination variation is not well understood outside of the complex species’ genomes, and it is particularly unclear in *Gossypium*. Cotton is the most important natural fibre crop and the second largest oil-seed crop. Here, we found that the genetic and physical maps distances did not have a simple linear relationship. Recombination rates were unevenly distributed throughout the cotton genome, which showed marked changes along the chromosome lengths and recombination was completely suppressed in the centromeric regions. Recombination rates significantly varied between A-subgenome (At) (range = 1.60 to 3.26 centimorgan/megabase [cM/Mb]) and D-subgenome (Dt) (range = 2.17 to 4.97 cM/Mb), which explained why the genetic maps of At and Dt are similar but the physical map of Dt is only half that of At. The translocation regions between A02 and A03 and between A04 and A05, and the inversion regions on A10, D10, A07 and D07 indicated relatively high recombination rates in the distal regions of the chromosomes. Recombination rates were positively correlated with the densities of genes, markers and the distance from the centromere, and negatively correlated with transposable elements (TEs). The gene ontology (GO) categories showed that genes in high recombination regions may tend to response to environmental stimuli, and genes in low recombination regions are related to mitosis and meiosis, which suggested that they may provide the primary driving force in adaptive evolution and assure the stability of basic cell cycle in a rapidly changing environment. Global knowledge of recombination rates will facilitate genetics and breeding in cotton.

## Introduction

Cotton (*Gossypium* spp.) is globally the primary nature fibre crop and the largest source of renewable plant-based fibre. In addition to fibre, the cotton seeds have distinctive uses and economic importance and provide a significant source of vegetable oil and high protein meals [1], which makes cotton an important food source for humans and livestock [2]. Recently, the genomes of *G*. *arboreum*, *G*. *raimondii*, *G*. *hirsutum* and *G*. *barbadense* have successively been sequenced [3–9], which has advanced the understanding of cotton genomics and genetics. Genetic maps also help us to understand the genetic makeup of the genome and to obtain the localization of genes of interest by analyzing genetic linkage with the mapped markers [10]. More than 30 marker-based genetic maps have been developed in cotton, and most of them are interspecific crosses between *G*. *hirsutum* and *G*. *barbadense*, including various kinds of markers [11].

Recombination plays a key role in biological evolution, and is central to the evolutionary success within eukaryotes. When crossover recombination occurs, the homologous chromosomes exchange genetic information, resulting in allele shuffling and generating novel genetic variation by breaking the associations between linked genes, but it may homogenize alleles through gene conversion [12–14]. Genetic variation arises and is separated by natural selection in genetic backgrounds, which shapes the adaptive evolution of organisms and indirectly shapes genome evolution.

Recently, recombination has been analysed by estimating genetic distance divided by the physical distance using linkage disequilibrium (LD) mapping and linkage mapping with molecular markers [15]. Generally, the classical approach for investigating recombination rate across the genome is to build a high-density genetic map and match it to the corresponding physical map, which can directly estimate the recombination rate between the genetic distance and the corresponding physical distance and is essential to understand the intergenerational variability of the genome [16].

Understanding variation in the recombination rate is not only fundamental to many aspects of genetics but also will help to gain a better comprehending of genome evolution. Knowing the genomic distribution of recombination rate can help to predict the potential quantity and breeding method of the population response to environmental change [15]. Specifically, characterizing the recombination rate will contribute to predict the degree of LD and target marker densities for genomic selection, which shows good promise of genomic selection in the future for both animal and plant breeding [17]. Practically, there are applications in finding genes of interest that are detected by linkage mapping or association genetic studies so that we can introduce favorable alleles via breeding [15,18, 19].

Determining recombination rate variation and distribution across the cotton genome has been constrained by the accuracy of existing genetic maps and the reference genome sequence. The genomic resources required to investigate the recombination rate for cotton have only recently become available due to genome sequencing [7, 20, 21]; thus, there is little knowledge of the genomic distribution of the recombination rate in the cotton genome. Fortunately, this provides us the opportunity to directly estimate the genome-wide recombination rate in cotton using a high-density genetic linkage map. Although a recent study has provided some information about recombination in cotton [20], there is still little information regarding to the genome-wide variation of recombination rate and its correlations with genomic features in cotton.

In our laboratory, a high-density genetic map was constructed that included 5,152 loci; the total length was 4696.03 cM, and the average marker interval was 0.91 cM [21]. Having this linkge map available and knowing the genome sequence of tetraploid cotton allow us to investigate the landscape of genome-wide variation in the recombination rate. In this study, we aimed to (i) explore the differences of recombination rate between two subgenomes, and among different chromosomes; (ii) to reveal the correlations between recombination rate and the genomic features, including density of genes, transposable elements and markers, and the distance from centromere; and (iii) to identify gene ontology enrichments in the hotspot versus coldspot regions.

## Materials and methods

### Marker sequence data

A total of 5,299 primer pairs were used and described in details by Li et al. [21]. The primer set included 4,569 SSRs (http://www.cottongen.org/) and 730 other types of primers [22, 23]. Using the Cottongen database [24], the potential intron polymorphism (PIP) database [22] and Cotton Marker database (CMD) (http://cottonmarker.org) [25], the sequences corresponding to these markers were found and downloaded. The CMD database has been superseded by CottonGen database because it is better for data retrieval, visualization, data sharing and data mining in cotton studies [24]. Some markers sequences that were not found via their ID numbers were excluded from the analysis in this study. A total of 4,807 available marker-derived sequences from the 5,152 mapped markers were used for downstream analysis.

### The relationship between genetic and physical maps

The high-density genetic map was 4696.03 cM in total length and 0.91 cM in mean distance [21]. Herein, to obtain the physical location of the markers, the 4,807 available nucleotide sequences were aligned back to TM-1 genomic sequence [7] using the automated batch BLASTN search with E ≤1e^−10^. The best hit was chosen for each marker to infer the map position combining adjacent markers’ positions. A custom Perl script was used to identify the actual marker’ positions relative to the TM-1 genomic sequence [7]. In addition, some markers that were not confirmed in terms of physical position were not included in the downstream analysis. Finally, 4,157 filtered markers were physical mapped and the physical map of each chromosome was created with the program ‘‘MapChart” [26]. The genome coverage of the physical map was calculated based on the reference genome size [7]. With the genetic and physical positions of markers, the collinearity of markers was compared and showed using the software Circos 0.67 [27].

### Genomic features distribution

Based on the previous studies [7, 20], the putative centromeric regions have been identified, except the A08 chromosome. The genomic distributions of the recombination rate, genes, transposable elements (TEs) and markers were investigated. All the nucleotide binding site (*NBS*)-leucine-rich repeat (*LRR*) genes were obtained from the TM-1 cotton genome [7]. The average recombination rate and the number of markers, genes and TEs in each 1Mb chromosomal region was calculated using a customized Perl script, which were showed with histogram and heat map using the Circos 0.67 software [27]. The correlations between average recombination rate and gene density, TE density, marker density and the distance from centromere were determined in each 1-Mb chromosomal region by the SPSS 17.0 software (SPSS, Chicago, USA).

### Estimation of recombination rates

Each marker’s start and end locations were averaged to determine the confirmed physical position in the genome [28]. After determining the physical positions of the markers and then combining the genetic positions, the local recombination rates were estimated along each of the 26 chromosomes using MareyMap [29]. The relationship of genetic and physical positions was demonstrated by a scatter plot with the markers’ genetic positions (cM) versus physical positions (Mb). The recombination map was constructed and displayed by a smooth line chart in non-overlapping 1-Mb windows with the Loess method via using MareyMap [29].

Each cotton chromosome was divided into the non-overlapping windows (window size was 100 kb) and the recombination rate of each window was calculated. Based on the frequency distribution of the recombinant rate, two extreme ranges (1%) were chosen as the thresholds, which were 27 cM/Mb and 0.05 cM/Mb. If the recombination rate was ≥ 27 cM/Mb, the high recombination rate windows were considered and the genes in this window were collected. Similarly, if the rate was ≤ 0.05 cM/Mb, the low recombination rate windows outside the centromeric regions were considered and the genes in this window were collected. The putative functions of the genes in the high and low recombination regions were analyzed using Fisher’s exact test in Blast2GO version 2.8 [30] with a p-value cut-off of ≤ 0.01.

## Results

### Overview of genetic map and construction of the physical map

The high-density genetic map that mentioned above with 5,152 loci was 4696.03 cM and the mean distance was 0.91 cM. The At subgenome was 2359.36 cM with 2,473 loci and the mean distance was 0.95 cM; whereas the Dt subgenome was 2336.67 cM with 2,679 loci and the mean distance was 0.87 cM.

Furthermore, in this study, after excluding markers with no corresponding sequences, 4,157 markers were matched on the 26 physical chromosomes, and the physical map was constructed with an average of 160 markers per chromosome. For the At and Dt subgenomes, there were 1,939 and 2,218 total markers with an average of 149 and 170 markers per chromosome, respectively (Table 1 and S1 Fig). In At, chromosome A05 contained 224 markers, which was the largest number of markers on any chromosome, whereas A04 only contained 97 markers. In Dt, the highest number of markers (235) was located on chromosome D05 and the lowest number of markers (118) was located on D04. The physically mapped markers on the chromosomes exhibited a higher marker density in Dt (2.86/Mb) than in At (1.69/Mb); the maximum marker density was on A05 (2.41/Mb) and D05 (3.79/Mb), and minimum was on A06 (1.14/Mb) and D02 (2.25/Mb; Table 1). In addition, 384 markers were identified on the cotton genome scaffolds. Among them, 348 markers were on the scaffolds that were not anchored to certain chromosomes, which included 191 markers in the At subgenome and 157 markers in the Dt subgenome. The remaining 36 markers were on non-chromosome anchored scaffolds (Table 1). The genome coverage of the physical map showed a drastic change along each chromosome, which ranged from 21.05% to 81.71% (Table 2). Generally, the genome coverage was higher on Dt chromosomes than on At chromosomes (Table 2).

**Table 1:** The physical distribution statistics of markers on 26 chromosomes in cotton.

Chromosome	Markers on chromosomes in physical map	Markers on scaffold	Unanchored markers	Marker density
A01	120	7	16	1.20
A02	100	12	18	1.19
A03	134	10	25	1.33
A04	97	11	14	1.54
A05	224	27	34	2.41
A06	119	16	30	1.14
A07	131	11	21	1.66
A08	155	23	35	1.49
A09	143	17	20	1.91
A10	136	8	36	1.35
A11	208	31	32	2.20
A12	201	18	22	2.28
A13	171	18	22	2.14
At subgenome	1939	209	325	1.68
D01	189	6	20	3.05
D02	153	6	21	2.25
D03	118	18	14	2.51
D04	118	12	17	2.27
D05	235	27	44	3.79
D06	194	20	24	2.52
D07	148	17	14	2.64
D08	208	4	13	3.15
D09	173	11	25	3.37
D10	147	18	18	2.26
D11	216	21	28	3.22
D12	164	5	25	3.23
D13	155	10	23	2.52
Dt subgenome	2218	175	286	2.83
Whole genome	4157	384	611	2.25

At: A-subgenome; Dt: D-subgenome; Marker density: the number of markers per Mb

**Table 2:** Summary of the integrated chromosome features and the correlations.

Chr	RR	CPL	CGL	Gene	TE	G-cov (%)	Correlations of RR and genomic features and distance from centromere			
							Gene	TE	Marker	DIS
A01	2.83	99.88	186.87	19.90	1415.09	34.53	0.62**	-0.29**	0.60**	0.46**
A02	2.69	83.45	156.03	20.50	1469.99	24.39	0.88**	-0.29**	0.71**	0.39**
A03	2.14	100.26	164.93	19.15	1404.40	34.66	0.72**	-0.25*	0.56**	0.39**
A04	2.61	62.91	149.82	20.41	1418.03	46.84	0.70**	-0.02	0.41**	0.36**
A05	3.16	92.05	242.76	38.68	1253.56	21.05	0.60**	-0.47**	0.46**	0.37**
A06	1.86	103.17	171.43	17.49	1423.33	22.54	0.45**	-0.12	0.42**	0.46**
A07	1.60	78.25	105.78	27.14	1361.58	31.27	0.34**	-0.13	0.36**	-0.26
A08	1.71	103.63	151.02	21.82	1420.45	29.11	0.57**	-0.23*	0.37**	-
A09	2.29	75.00	148.83	29.40	1362.67	44.72	0.63**	-0.54**	0.43**	0.55**
A10	2.81	100.87	200.04	21.61	1401.38	26.36	0.39**	-0.24*	0.18	0.36**
A11	3.26	93.32	234.77	31.23	1317.03	28.62	0.52**	-0.32**	0.46**	0.41**
A12	3.04	87.48	238.05	28.48	1358.11	34.21	0.65**	-0.45**	0.40**	0.35**
A13	3.26	79.96	208.14	25.66	1423.89	34.23	0.73**	-0.43**	0.53**	0.54**
At	2.55	89.25	181.42	24.56	1386.82	31.07	0.59**	-0.30**	0.45**	0.31**
D01	3.98	61.46	197.10	36.39	1219.35	41.69	0.59**	-0.04	0.42**	0.44**
D02	2.87	67.28	164.12	34.91	1251.91	37.40	0.64**	-0.02	0.32**	0.46**
D03	4.44	46.69	162.23	35.49	1187.89	81.71	0.77**	0.14	0.59**	0.37**
D04	4.17	51.45	169.93	36.73	1266.04	41.71	0.51**	-0.02	0.36**	0.47**
D05	4.36	61.93	252.27	60.05	1027.06	26.01	0.57**	-0.49**	0.46**	0.59**
D06	2.77	64.29	172.19	34.80	1241.00	33.75	0.78**	0.14	0.50**	0.50**
D07	2.97	55.31	94.32	42.02	1198.14	51.94	0.58**	-0.05	0.40**	0.37**
D08	3.16	65.89	198.85	39.92	1239.15	38.08	0.72**	-0.26*	0.38**	0.54**
D09	3.63	51.00	193.19	47.22	1158.47	71.25	0.43**	-0.37**	0.01	0.40**
D10	2.17	63.37	117.61	37.29	1185.40	48.84	0.56**	-0.17	0.20	0.46**
D11	4.97	66.09	256.03	49.58	1138.27	32.32	0.50**	-0.14	0.35**	0.42**
D12	4.21	59.11	211.90	43.80	1160.78	49.67	0.66**	-0.21	0.52**	0.51**
D13	2.94	60.53	146.95	40.30	1217.23	44.41	0.49**	-0.06	0.61**	0.51**
Dt	3.56	59.57	179.74	41.51	1193.23	44.80	0.57**	-0.15**	0.40**	0.43**
WG	2.13	74.41	180.58	31.36	1309.17	36.56	0.58**	-0.25**	0.43**	0.30**

RR: Recombination rate (cM/Mb); CPL: Chromosome physical length (Mb); CGL: Chromosome genetic length (cM) [12]; Gene: Gene density, the number of genes per Mb; TE: Transposable element density, the number of transposable elements per Mb; G-cov: the genome coverage of the physical map; DIS: The distance from centromere; At: A-subgenome level; Dt: D-subgenome level; WG: whole genome wide level;

* Correlation is significant at the 0.05 level;

** Correlation is significant at the 0.01 level;

### Collinearity of the genetic and physical maps

**Fig 1:**
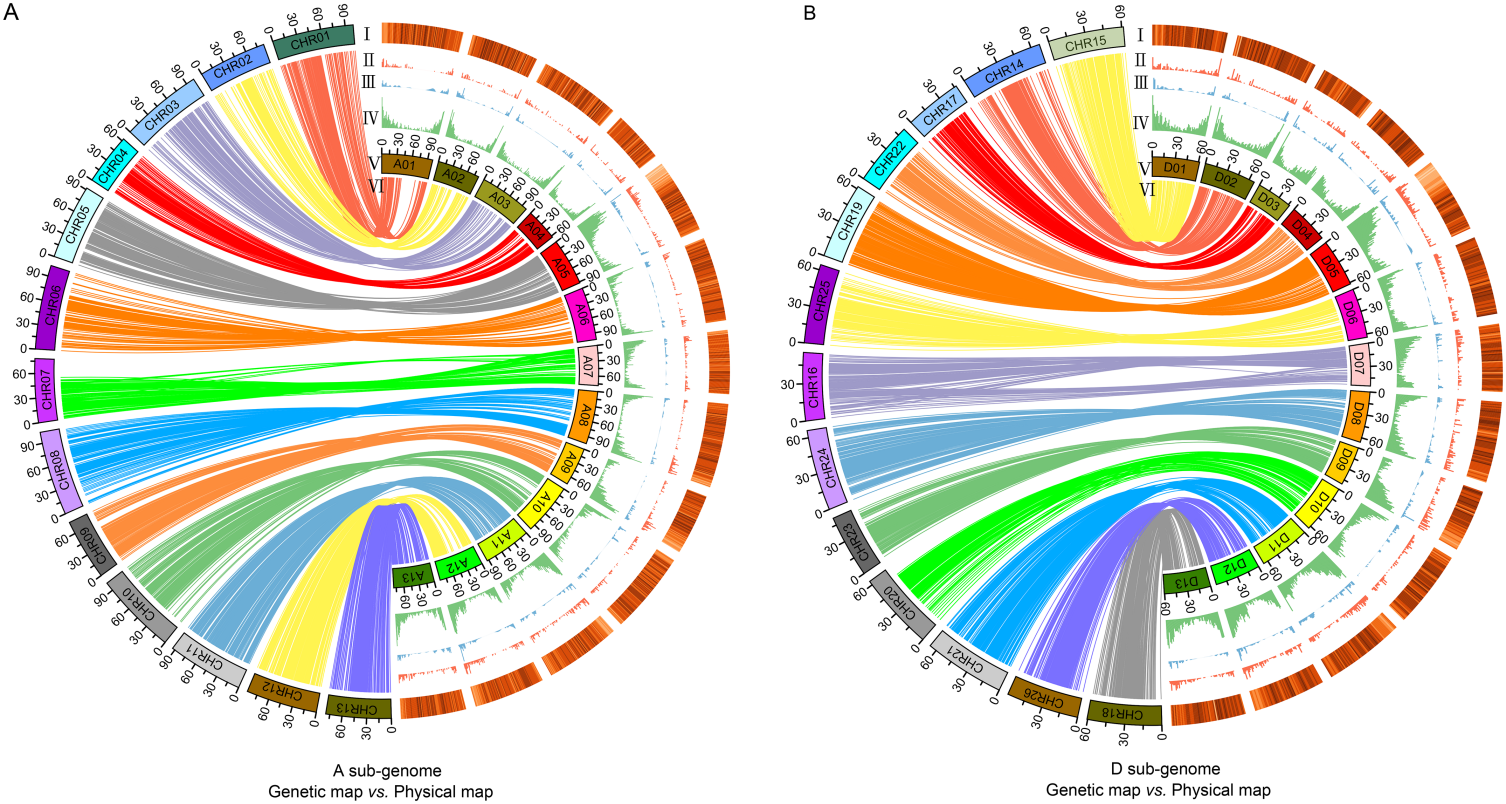
Collinearity of the genetic and physical maps and genomic distribution of TEs, markers, recombination rate and genes on each chromosome. Collinearity of the genetic and physical maps and genomic distribution of TEs, markers, recombination rate and genes on each chromosome in At (A) and Dt (B). (A, B)I-IV: The genomic distribution of TEs, markers, recombination rate and genes on each chromosome are shown in heat map, red bar graphs, blue bar graphs and green bar graphs;V: The chromosomes;VI: Collinearity of the genetic and physical maps.

**Fig 2:**
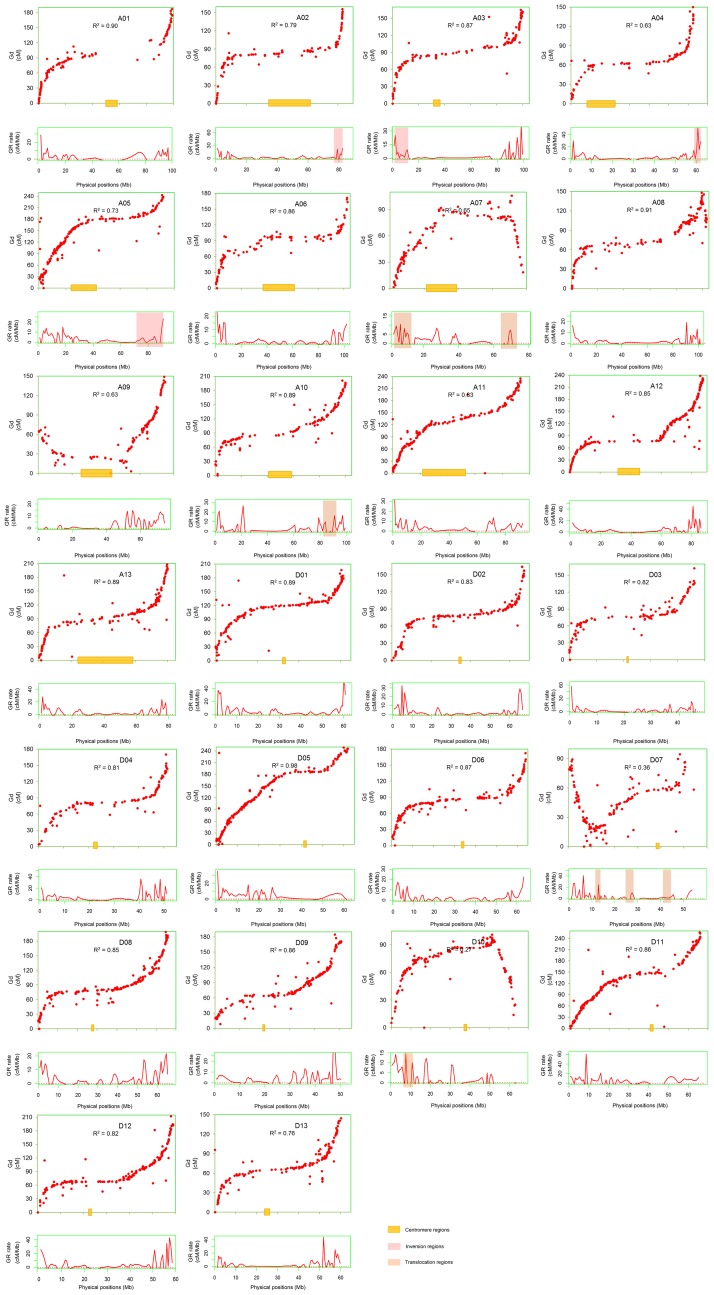
The correlation of genetic and physical maps, the estimated local recombination rates and its distribution in chromosomal rearrangement regions. The red dots represent the genetic and physical positions of markers. The red curves below the scatter plots of SSRs represent the estimated local recombination rates. The value of R^2^ represent the correlation between the genetic and physical maps. Shadow represents the centromere regions, inversion regions and translocation regions.

The collinearity of the high-density genetic and physical maps shown in Fig 1A(VI) and 1B(VI), was investigated on various chromosomes, At and Dt subgenomes, respectively. The results indicated that the physical map that was constructed by these markers was very effective. Most of the chromosomal markers had highly collinearity between the genetic and physical maps, especially on chromosomes D05, A08 and A01. Chromosomes A02, A05, A07, A09 and D13 were moderately consistent with their physical locations (Fig 2). Chromosomes D07 and D10 showed serious disagreement between the genetic and physical maps on either side of the chromosomes, which showed that the large segments on the two chromosomes were not co-linear, in terms of the physical map (Fig 2). In addition, the Spearman correlation coefficient between the genetic and physical positions of each chromosome was generally consistent with the levels of genetic collinearity. The value of Spearman correlation coefficient on chromosome D05 was 0.98, which indicated a strong correlation (Fig 2). The Dt subgenome exhibited better compatibility with the physical map than At, except chromosome D07 and D10 (Figs 1BVI and 2). The homologous chromosomes A01 and D01; along A08 and D08 showed high Spearman correlation coefficients (Fig 2). In contrast, there were low Spearman correlation coefficients in the homologous chromosomes A07 and D07 (Fig 2). At the whole genome level, the genetic and physical map distances did not have a simple linear relationship (Fig 2).

### Landscape of genome-wide recombination rate

The landscape of the genome-wide variation of the recombination rate is shown in Fig 2 and exhibited an informative and precise estimate of the recombination rate per physical distance (Mb) along each of the 26 chromosomes at the sub-regional level. The distribution of the average genome-wide recombination rate was non-random, and the recombination rate in the distal chromosomal regions was higher than that of the proximal regions (Fig 1III). Recombination rates also varied across individual chromosomes, as illustrated in Fig 2. Although most of the chromosomes’ proximal regions lack recombination, some recombination spikes were stored in the centromeric and pericentromeric regions on a few chromosomes, such as A05 and A07 (Fig 2). In addition to the centromeric and pericentromeric regions, there were some other locations where little to no recombination occurred, such as the homologous chromosomes A07 and D07 as well as A09 and D09, especially in the left arm of A09 and right arm of D07 (Figs 1III and 2).

The genome-wide average recombination rate significantly varied along each chromosome, which ranged from 1.60 cM/Mb to 4.97 cM/Mb (Table 2). In At, chromosomes A06, A07 and A08 had a lower average recombination rate, which was 1.86 cM/Mb, 1.60 cM/Mb and 1.71 cM/Mb, respectively. In contrast, higher recombination rates were found on chromosomes A05 (3.16 cM/Mb), A11 (3.26 cM/Mb) and A13 (3.26 cM/Mb; Table 2). In Dt, lower recombination rates were on chromosomes D10 (2.17 cM/Mb), D06 (2.77 cM/Mb) and D07 (2.97 cM/Mb), and higher recombination rates were on chromosomes D03 (4.44 cM/Mb), D05 (4.36 cM/Mb) and D11 (4.97 cM/Mb). Obviously, the average recombination rates of D03 and D11 were more than twice as high as those of A07 and A08. Generally, the recombination rate was higher on Dt chromosomes than on At chromosomes (Table 2). The homologous chromosomes A03 and D03 demonstrated that they had similar genetic length (164.93 cM and 162.23 cM, respectively), but the physical length of chromosome D03 (46.69 Mb) was less than half of the physical length of A03 (100.08 Mb), and the recombination rate of D03 (4.44 cM/Mb) was two times higher than that of A03 (2.14 cM/Mb). Similarly, the homologous chromosomes A06 and D06 have almost the same genetic length (171.43 cM and 172.19 cM, respectively), but the chromosome physical length of A06 (103.17 Mb) was 1.60 times higher than that of D06 (64.29 Mb), and the recombination rate of D06 (2.77 cM/Mb) was 1.49 times higher than that of A06 (1.86 cM/Mb; Table 2). The homologous chromosomes A07 and D07 showed that the genetic length of A07 (105.78 cM) was 1.12 times higher than that of D07 (94.32 cM), but the recombination rates of D07 (2.97 cM/Mb) was 1.86 times higher than that of A07 (1.60 cM/Mb), and the physical length of chromosome A07 (78.25 Mb) was 1.44 times higher than that of D07 (54.31 Mb) (Table 2). Comparative analysis of the homologous chromosomes A13 and D13 found that the genetic length, chromosome physical length and recombination rate of A13 were 1.41, 1.32 and 1.11 times higher than those of D13, respectively (Table 2). The whole genome-wide average recombination rate was 2.13 cM/Mb, and it was higher in Dt (3.56 cM/Mb) than in At (2.55 cM/Mb; Table 2). Although the average genetic length of At (181.42 cM) and Dt (179.74 cM) were similar, the physical length of Dt chromosomes was half as short as At chromosomes, and the recombination rates were nearly 0.5 times higher on Dt chromosomes.

Chromosomal rearrangement may have a considerable influence on recombination. The translocation regions between A02 and A03 and between A04 and A05 and inversion regions on A10, D10, A07 and D07 were observed with the physical markers locations, which indicated that the chromosomal translocation and inversion that occurred in the distal regions of the chromosomes had a relatively high recombination rate (Fig 2).

### Correlations between recombination rate and genomic features

The distributions of recombination rate, genes, TEs and markers were not highly uniform along individual chromosome’s length. The density of recombination rate, genes and markers decreased towards the chromosomes’ middles; while, TE density greatly increased towards the centromeric regions from both chromosomes arms (Fig 1I–1IV). On the whole genome level, the recombination rate was significant positively correlated with the density of genes (R^2^ = 0.58; *p* ≤ 0.01) and markers (R^2^ = 0.43; *p* ≤ 0.01), but significant negatively correlated with the density of transposable elements (R^2^ = -0.25; *p* ≤ 0.01; Table 2). The recombination rate showed a significant positive correlation with the distance from the centromere (R^2^ = 0.30; *p* ≤ 0.01). The correlations of recombination rate and genomic features showed a similar varying tendency between At and Dt, and the average correlation coefficient of recombination rate and distance from the centromere on Dt (R^2^ = 0.43; *p* ≤ 0.01) was higher than that of At (R^2^ = 0.31; *p* ≤ 0.01; Table 2). The homologous chromosomes A03 (R^2^ = 0.72; *p* ≤ 0.01) and D03 (R^2^ = 0.77; *p* ≤ 0.01) showed a relatively high positive correlation between recombination rate and gene density. There was a higher negative correlation on homologous chromosomes A05 (R^2^ = -0.47; *p* ≤ 0.01) and D05 (R^2^ = -0.49; *p* ≤ 0.01), and A09 (R^2^ = -0.54; *p* ≤ 0.01) and D09 (R^2^ = -0.37; *p* ≤ 0.01) than others between recombination rate and TEs density. Taking individual chromosome into account, A02 had a strong relation between recombination rate and gene density (R^2^ = 0.88; *p* ≤ 0.01); however, A07 showed a lowest correlation (R^2^ = 0.39; *p* ≤ 0.01; Table 2). A09 showed a significant negative relation between recombination rate and TEs density (R^2^ = -0.54; *p* ≤ 0.01); while, D03 and D06 showed weakly positive correlation (R^2^ = 0.14). The correlation between recombination rate and marker density demonstrated drastic differences in different chromosomes, with correlation coefficient R^2^ value ranging 0.01 (D09) to 0.71 (A02; Table 2). However, recombination rate showed a positive correlation with the distance from the centromere, except A07 (R^2^ = -0.26) and D08 (unavailable). D05 showed the highest correlation coefficient (R^2^ = 0.59; Table 2).

### Functional categories of the genes in high or low recombination regions

**Fig 3:**
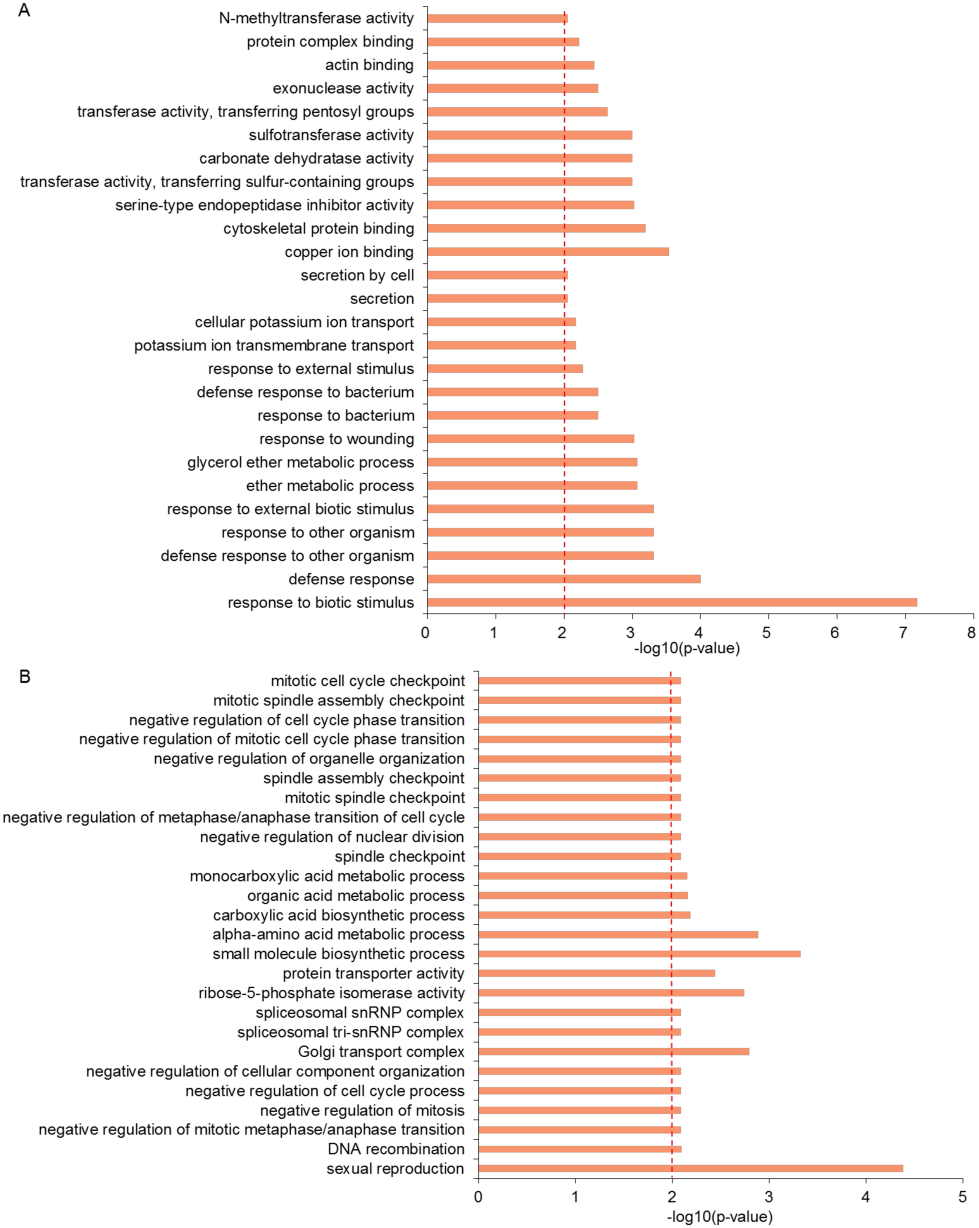
The GO terms distribution of the genes in the high and low recombination regions. The GO terms distribution of the genes in the high recombination regions (A) and the low recombination regions (B).

To survey the functional categories of genes located in the high and low recombination regions, the genes were classed by the GO. The significantly enriched results of GO revealed that most of the genes in the regions with high recombination rates had putative functions responding to the environmental stimulus. For instance, response to biotic stimulus, defense response, response to wounding, response to bacterium, defense response to bacterium, response to external stimulus, response to external biotic stimulus, etc., were response to environmental stimuli (Fig 3A). In contrast, the results of GO in the low recombination regions showed that the genes were related to the spindles and mitosis, for example, spindle checkpoint, mitotic spindle checkpoint, mitotic cell cycle checkpoint, negative regulation of cell cycle process, sexual reproduction and negative regulation of mitosis, etc. (Fig 3B and S1 Fig).

**Fig 4:**
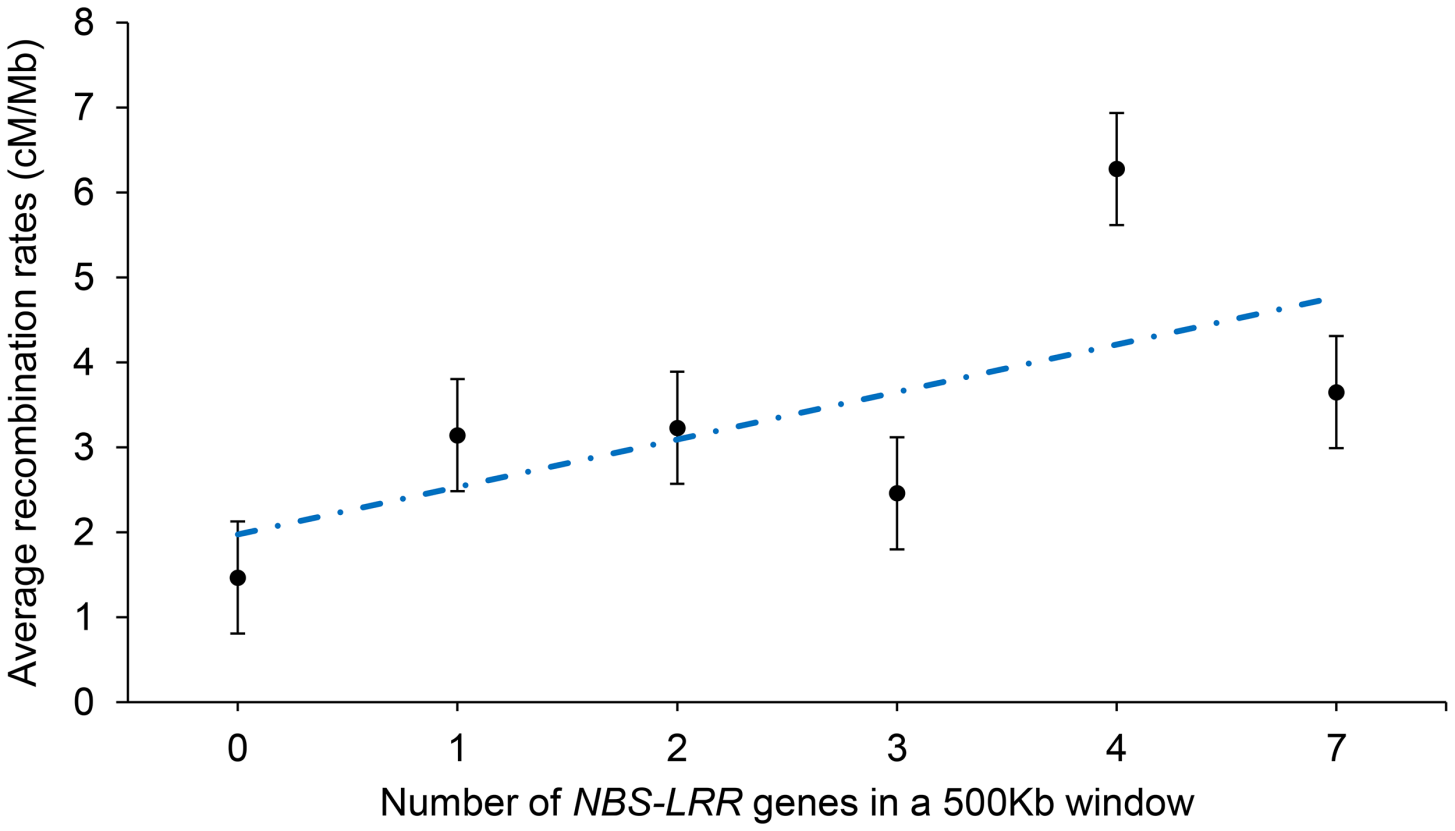
The linear relationship between recombination rates and density of *NBS-LRR* genes. The linear relationship between recombination rates and density of *NBS-LRR* genes. Error bars represent ± standard error.

In addition, based on the existing data, a positive correlation was found between recombination rates and the density of the *NBS*-*LRR* genes in cotton genome which are related to disease resistance (Fig 4).

## Discussion

In this study, based on the high-density genetic map containing 5,152 loci [21] and the published TM-1 cotton genomic sequence [7], the corresponding physical map, including 4,157 markers, was constructed (S1 Fig). Chromosomes A05 and D05 had the highest marker density according to the physical map, and chromosomes A06 and D02 had the lowest marker density (Table 1). Chromosome D03 showed the highest genome coverage compared to other chromosomes (Table 2). The recombination rate, genes and markers were positively related and showed uneven distribution across various chromosomes, with their densities increasing towards the chromosomes’ ends rather than TEs (Fig 1I–1IV).

Recombination, as a vital component in crop breeding and genetics, plays a key role in genomic evolution, domestication and the improvement of crops [31, 32], and can improve crops by creating genetic variation in gametes and new combinations of available genes. Understanding chromosomal recombination rate distribution is very important for characterizing and cloning genes. The distribution of recombination rate showed a close association with genes/markers distribution on cotton chromosomes (Figs 1I–1IV and 2). However, not all the non-random recombination distributions could be explained by the gene density [33–35], chromosomal inversion may cause the inactivation of recombination. Compared with the centromeric regions, high recombination rate was observed in the telomeric regions (Figs 1III and 2), which demonstrated that recombination was suppressed in the centromeric regions. Similarly, in maize, the recombination rates were highest towards the telomeric ends of the chromosomes and highly suppressed near the centromeres [36, 37].

Recombination greatly varied across the length of cotton chromosomes, and the correlation of recombination with the distance from the centromere of chromosomes (R^2^ = 0.30; *p* ≤ 0.01) showed a higher correlation than soybean [28]. However, chromosome A07 showed a negative correlation between them (R^2^ = -0.26; Table 2), which may be due to the repeated inversions causing a significant change in recombination rate [21, 38]. The correlation between recombination rate and the distance from the chromosome centromere showed a great difference in different genome species, for example, the larger genomes like barley and wheat were known to show a higher correlation [39, 40], but the smaller genomes like soybean and *Arabidopsis* showed the relatively lower correlation, especially in *Arabidopsis* [28, 41]. An exponential increase between recombination rates and distance from the centromere was found in wheat [41]. In soybean, recombination had a relatively weak correlation relative to the distance from the centromere [28]. However, recombination showed very little correlation with the distance from the centromere in the model plant *Arabidopsis* [42].

The cotton genome is approximately 2 times bigger than the soybean genome and 16 times bigger than *Arabidopsis*, but it is approximately 7 times smaller than the wheat genome [43]. The distribution of recombination and gene was different between cotton and other genomic species such as soybean, barley and wheat, which may be due to the differences in genome sizes [44]. Moreover, chromosomal rearrangement could cause a significant divergence during the evolutionary process in the genomic regions of two different rice subspecies, which could lead to recombination rate changes [38]. The previous study revealed that inversions occurred between the cotton homologous chromosomes A07 and D07 as well as between A10 and D10. Translocations occurred between A02 and A03 and between A04 and A05 in cotton [21]. In this study, it was found that chromosomal rearrangement in these regions may cause the activation of recombination in distal regions of the chromosomes (Fig 2).

Furthermore, recombination hotspot and coldspot regions were observed on all the chromosomes. Previous studies showed that the recombination hotspot regions had small areas of chromosome length, but occupied large areas of genetic length, which indicated uneven recombination distribution [28, 34]. The correlation between recombination rate and the distance from centromere was not very high, which may be due to the unevenness generated by hotspots and coldspots of recombination. Even there was barely any recombination in the distal regions of chromosomes that contained large chromosomal areas. The results may explain why the average genetic length of At (181.42 cM) and Dt (179.74 cM) were similar, but the Dt chromosomes were half as short as the At chromosomes, and the recombination rates were nearly 0.5 times higher on Dt chromosomes.

The genes responding to environmental stimuli usually have the higher recombination rates [45–47]. The significant GO enrichment result of the genes in the high recombination rates regions showed that many genes had functions responding to the environmental stimulus compared to those in the low recombination regions, for instance, response to biotic stimulus, response to wounding, response to bacterium, defense response to bacterium, response to external stimulus, etc. (Fig 3A), suggesting that these genes may respond to environmental stimuli and play an important role in adapting to the rapidly changing environments [47]. On the contrary, the genes in the low recombination regions were mostly related to mitotic spindle checkpoint, negative regulation of cell cycle process and negative regulation of mitosis, etc. (Fig 3B), which are important for mitosis and meiosis. The low recombination can assure the stability of basic cell cycle.

The *NBS-LRR* genes, as the largest group of plant disease resistance genes, were involved in the process of pathogen recognition and led to disease resistance [46]. Combining with previous researches [45, 47], it has been found that the clustered *NBS-LRR* genes showed higher recombination rates than singleton *NBS-LRR* genes. As shown in Fig 4, there was a positive correlation between the density of *NBS-LRR* genes and recombination rates, suggesting that these stress-responsive genes may improve the possibility of retention, and frequent recombination may be very important to adapt to a complex environment [45, 47].

## Conclusions

Combing the genetic and physical maps makes it possible to shed light on the characteristics of recombination in cotton. The recombination rates were significantly different between At and Dt subgenomes and among different chromosomes. The translocation regions between A02 and A03 and between A04 and A05, and inversion regions on A10, D10, A07 and D07 showed a relatively high rate of recombination. The relationship between recombination rate and the genomic features, including density of genes, markers, and the distance from centromere, demonstrated a positive correlation as compared to transposable elements. The results of GO enrichments in the hotspot and coldspot regions showed that these genes respond to environmental stimuli and are related to mitosis and meiosis, respectively. Overall, the observations in this study provide insights into recombination rate variations, which will facilitate the understanding of cotton genetics.

## Supporting information

S1 Fig The markers’ physical map. (PDF)

S1 Table All the GO terms distribution of the genes in the low recombination regions. (XLS)

## References

[1] Gore MA, Fang DD, Poland JA, Zhang J, Percy RG, Cantrell RG, et al Linkage map construction and quantitative trait locus analysis of agronomic and fiber quality traits in cotton. The Plant Genome. 2014;7(1):1–10.

[2] Sunilkumar G, Campbell LM, Puckhaber L, Stipanovic RD, Rathore KS. Engineering cottonseed for use in human nutrition by tissue-specific reduction of toxic gossypol. Proc Natl Acad Sci. 2006;103(48):18054–18059. doi: 10.1073/pnas.0605389103 17110445 10.1073/pnas.0605389103 PMC1838705

[3] Li FG, Fan GY, Wang KB, Sun FM, Yuan YL, Song GL, et al Genome sequence of the cultivated cotton *Gossypium arboreum*. Nat Genet. 2014;46(6):567–572. doi: 10.1038/ng.2987 24836287 10.1038/ng.2987

[4] Wang KB, Wang ZW, Li FG, Ye WW, Wang JY, Song GL, et al The draft genome of a diploid cotton *Gossypium raimondii*. Nat Genet. 2012;44(10):1098–1103. doi: 10.1038/ng.2371 22922876 10.1038/ng.2371

[5] Paterson AH, Wendel JF, Gundlach H, Guo H, Jenkins J, Jin DC, et al Repeated polyploidization of *Gossypium* genomes and the evolution of spinnable cotton fibres. Nature. 2012;492(7429):423–427. doi: 10.1038/nature11798 23257886 10.1038/nature11798

[6] Li FG, Fan GY, Lu CR, Xiao GH, Zou CS, Kohel RJ, et al Genome sequence of cultivated Upland cotton (*Gossypium hirsutum* TM-1) provides insights into genome evolution. Nat Biotechnol. 2015;33(5):524–U242. doi: 10.1038/nbt.3208 25893780 10.1038/nbt.3208

[7] Zhang T, Hu Y, Jiang W, Fang L, Guan X, Chen J, et al Sequencing of allotetraploid cotton (*Gossypium hirsutum* L. acc. TM-1) provides a resource for fiber improvement. Nat Biotechnol. 2015;33(5):531–537. doi: 10.1038/nbt.3207 25893781 10.1038/nbt.3207

[8] Yuan D, Tang Z, Wang M, Gao W, Tu L, Jin X, et al The genome sequence of Sea-Island cotton (*Gossypium barbadense*) provides insights into the allopolyploidization and development of superior spinnable fibres. Sci Rep. 2015;5:17662 doi: 10.1038/srep17662 26634818 10.1038/srep17662 PMC4669482

[9] Liu X, Zhao B, Zheng HJ, Hu Y, Lu G, Yang CQ, et al *Gossypium barbadense* genome sequence provides insight into the evolution of extra-long staple fiber and specialized metabolites. Sci Rep. 2015;5:14139 doi: 10.1038/srep14139 26420475 10.1038/srep14139 PMC4588572

[10] Bernatzky R, Tanksley SD. Toward a saturated linkage map in tomato based on isozymes and random cDNA sequences. Genetics. 1986;112(4):887–898. 17246322 10.1093/genetics/112.4.887 PMC1202783

[11] Khan MK, Chen H, Zhou Z, Ilyas MK, Wang X, Cai X, et al Genome wide SSR high density genetic map construction from an interspecific cross of *Gossypium hirsutum* x *Gossypium tomentosum*. Front Plant Sci. 2016;7:436 doi: 10.3389/fpls.2016.00436 27148280 10.3389/fpls.2016.00436 PMC4829609

[12] Webster MT, Hurst LD. Direct and indirect consequences of meiotic recombination: implications for genome evolution. Trends Genet. 2012;28(3): 101–109. doi: 10.1016/j.tig.2011.11.002 22154475 10.1016/j.tig.2011.11.002

[13] Sun P, Zhang R, Jiang Y, Wang X, Li J, Lv H, et al Assessing the patterns of linkage disequilibrium in genic regions of the human genome. Febs J. 2011;278(19):3748–3755. doi: 10.1111/j.1742-4658.2011.08293.x 21824289 10.1111/j.1742-4658.2011.08293.x

[14] Chen JM, Cooper DN, Chuzhanova N, Ferec C, Patrinos GP. Gene conversion: mechanisms, evolution and human disease. Nat Rev Genet. 2007;8(10):762–775. doi: 10.1038/nrg2193 17846636 10.1038/nrg2193

[15] Gion JM, Hudson CJ, Lesur I, Vaillancourt RE, Potts BM, Freeman JS. Genome-wide variation in recombination rate in *Eucalyptus*. BMC Genomics. 2016;17. 10.1186/s12864-016-2884-y PMC4979139 27507140

[16] Kong A, Gudbjartsson DF, Sainz J, Jonsdottir GM, Gudjonsson SA, Richardsson B, et al A high-resolution recombination map of the human genome. Nat Genet. 2002;31(3):241–247. doi: 10.1038/ng917 12053178 10.1038/ng917

[17] Heffner EL, Sorrells ME, Jannink J-L. Genomic selection for crop improvement. Crop Sci. 2009;49(1):1.

[18] Goddard ME, Hayes BJ: Mapping genes for complex traits in domestic animals and their use in breeding programmes. Nat Rev Genet. 2009;10(6):381–391. doi: 10.1038/nrg2575 19448663 10.1038/nrg2575

[19] Rodgersmelnick E, Bradbury PJ, Elshire RJ, Glaubitz JC, Acharya CB, Mitchell SE, et al Recombination in diverse maize is stable, predictable, and associated with genetic load. Proc Natl Acad Sci. 2015;112(12):3823–3828. doi: 10.1073/pnas.1413864112 25775595 10.1073/pnas.1413864112 PMC4378432

[20] Wang S, Chen JD, Zhang WP, Hu Y, Chang LJ, Fang L, et al Sequence-based ultra-dense genetic and physical maps reveal structural variations of allopolyploid cotton genomes. Genome Biol. 2015;16. 10.1186/s13059-015-0678-1 PMC4469577 26003111

[21] Li X, Jin X, Wang H, Zhang X, Lin Z. Structure, evolution, and comparative genomics of tetraploid cotton based on a high-density genetic linkage map. DNA Res. 2016;23(3):283–293. doi: 10.1093/dnares/dsw016 27084896 10.1093/dnares/dsw016 PMC4909315

[22] Yang L, Jin G, Zhao X, Zheng Y, Xu Z, Wu W. PIP: a database of potential intron polymorphism markers. Bioinformatics. 2007;23(16):2174–2177. doi: 10.1093/bioinformatics/btm296 17545179 10.1093/bioinformatics/btm296

[23] Van Deynze A, Stoffel K, Lee M, Wilkins TA, Kozik A, Cantrell RG, et al Sampling nucleotide diversity in cotton. BMC Plant Biol. 2009;9. 10.1186/1471-2229-9-125 PMC2771027 19840401

[24] Yu J, Jung S, Cheng CH, Ficklin SP, Lee T, Zheng P, et al CottonGen: a genomics, genetics and breeding database for cotton research. Nucleic Acids Res. 42, D1229–1236. doi: 10.1093/nar/gkt1064 24203703 10.1093/nar/gkt1064 PMC3964939

[25] Blenda A, Scheffler J, Scheffler B, Palmer M, Lacape JM, Yu JZ, et al CMD: a Cotton Microsatellite Database resource for *Gossypium* genomics. BMC Genomics. 2006;7:132 doi: 10.1186/1471-2164-7-132 16737546 10.1186/1471-2164-7-132 PMC1539020

[26] Voorrips RE. MapChart: software for the graphical presentation of linkage maps and QTLs. J Hered. 2002;93(1):77–78. 12011185 10.1093/jhered/93.1.77

[27] Krzywinski M, Schein J, Birol I, Connors J, Gascoyne R, Horsman D, et al Circos: An information aesthetic for comparative genomics. Genome Res. 2009;19(9):1639–1645. doi: 10.1101/gr.092759.109 19541911 10.1101/gr.092759.109 PMC2752132

[28] Ott A, Trautschold B, Sandhu D. Using microsatellites to understand the physical distribution of recombination on soybean chromosomes. PLoS One. 2011;6(7):e22306 doi: 10.1371/journal.pone.0022306 21799819 10.1371/journal.pone.0022306 PMC3140510

[29] Rezvoy C, Charif D, Gueguen L, Marais GA. MareyMap: an R-based tool with graphical interface for estimating recombination rates. Bioinformatics. 2007;23(16):2188–2189. doi: 10.1093/bioinformatics/btm315 17586550 10.1093/bioinformatics/btm315

[30] Conesa A, Gotz S, Garcia-Gomez JM, Terol J, Talon M, Robles M. Blast2GO: a universal tool for annotation, visualization and analysis in functional genomics research. Bioinformatics. 2005;21(18):3674–3676. doi: 10.1093/bioinformatics/bti610 16081474 10.1093/bioinformatics/bti610

[31] Coop G, Przeworski M. An evolutionary view of human recombination. Nat Rev Genet. 2007;8(1):23–34. doi: 10.1038/nrg1947 17146469 10.1038/nrg1947

[32] Baudat F, Imai Y, de Massy B. Meiotic recombination in mammals: localization and regulation. Nat Rev Genet. 2013;14(11):794–806. doi: 10.1038/nrg3573 24136506 10.1038/nrg3573

[33] Liu S, Yeh CT, Ji T, Ying K, Wu H, Tang HM, et al Mu transposon insertion sites and meiotic recombination events co-localize with epigenetic marks for open chromatin across the maize genome. Plos Genet. 2009;5(11):e1000733 doi: 10.1371/journal.pgen.1000733 19936291 10.1371/journal.pgen.1000733 PMC2774946

[34] Walling JG, Shoemaker R, Young N, Mudge J, Jackson S. Chromosome-level homeology in paleopolyploid soybean (*Glycine max*) revealed through integration of genetic and chromosome maps. Genetics. 2006;172(3):1893–1900. doi: 10.1534/genetics.105.051466 16361231 10.1534/genetics.105.051466 PMC1456260

[35] Schmutz J, Cannon SB, Schlueter J, Ma JX, Mitros T, Nelson W, et al Genome sequence of the palaeopolyploid soybean. Nature. 2010;463(7278):178–183. doi: 10.1038/nature08670 20075913 10.1038/nature08670

[36] Chen MS, Presting G, Barbazuk WB, Goicoechea JL, Blackmon B, Fang FC, et al An integrated physical and genetic map of the rice genome. Plant Cell. 2002;14(3):537–545. doi: 10.1105/tpc.010485 11910002 10.1105/tpc.010485 PMC150577

[37] Schnable PS, Ware D, Fulton RS, Stein JC, Wei FS, Pasternak S, et al The B73 maize genome: complexity, diversity, and dynamics. Science. 2009;326(5956):1112–1115. doi: 10.1126/science.1178534 19965430 10.1126/science.1178534

[38] Wu J, Mizuno H, Hayashi-Tsugane M, Ito Y, Chiden Y, Fujisawa M, et al Physical maps and recombination frequency of six rice chromosomes. Plant J. 2003;36(5):720–730. 14617072 10.1046/j.1365-313x.2003.01903.x

[39] Kunzel G, Korzun L, Meister A. Cytologically integrated physical restriction fragment length polymorphism maps for the barley genome based on translocation breakpoints. Genetics. 2000;154(1):397–412. 10628998 10.1093/genetics/154.1.397 PMC1460903

[40] Akhunov ED, Goodyear AW, Geng S, Qi LL, Echalier B, Gill BS, et al The organization and rate of evolution of wheat genomes are correlated with recombination rates along chromosome arms. Genome Res. 2003;13(5):753–763. doi: 10.1101/gr.808603 12695326 10.1101/gr.808603 PMC430889

[41] Akhunov ED, Akhunova AR, Anderson OD, Anderson JA, Blake N, Clegg MT, et al Nucleotide diversity maps reveal variation in diversity among wheat genomes and chromosomes. BMC Genomics. 2010;11. 10.1186/1471-2164-11-702 PMC3022916 21156062

[42] Drouaud J, Camilleri C, Bourguignon PY, Canaguier A, Berard A, Vezon D, et al Variation in crossing-over rates across chromosome 4 of *Arabidopsis thaliana* reveals the presence of meiotic recombination "hot spots". Genome Res. 2006;16(1):106–114. doi: 10.1101/gr.4319006 16344568 10.1101/gr.4319006 PMC1356134

[43] Arumuganathan K, Earle ED. Nuclear DNA content of some important plant species. Plant Mol Biol Rep. 1991;9(3):208–218.

[44] Saintenac C, Falque M, Martin OC, Paux E, Feuillet C, Sourdille P. Detailed recombination studies along chromosome 3B provide new insights on crossover distribution in wheat (*Triticum aestivum* L.). Genetics. 2009;181(2):393–403. doi: 10.1534/genetics.108.097469 19064706 10.1534/genetics.108.097469 PMC2644935

[45] Yang SH, Gu TT, Pan CY, Feng ZM, Ding J, Hang YY, et al Genetic variation of *NBS-LRR* class resistance genes in rice lines. Theor Appl Genet. 2008;116(2):165–177. doi: 10.1007/s00122-007-0656-4 17932646 10.1007/s00122-007-0656-4

[46] Yang SH, Li J, Zhang XH, Zhang QJ, Huang J, Chen JQ, et al Rapidly evolving R genes in diverse grass species confer resistance to rice blast disease. Proc Natl Acad Sci. 2013;110(46):18572–18577. doi: 10.1073/pnas.1318211110 24145399 10.1073/pnas.1318211110 PMC3831948

[47] Si W, Yuan Y, Huang J, Zhang X, Zhang Y, Zhang Y, et al Widely distributed hot and cold spots in meiotic recombination as shown by the sequencing of rice F_2_ plants. New Phytol. 2015;206(4):1491–1502. doi: 10.1111/nph.13319 25664766 10.1111/nph.13319

