# Supplementary figures and images for: Genome-wide recombination rate variation in a recombination map of cotton
Source: PLoS One. 2017 Nov 27;12(11):e0188682. doi: 10.1371/journal.pone.0188682 (PMC5703465; doi:10.1371/journal.pone.0188682)

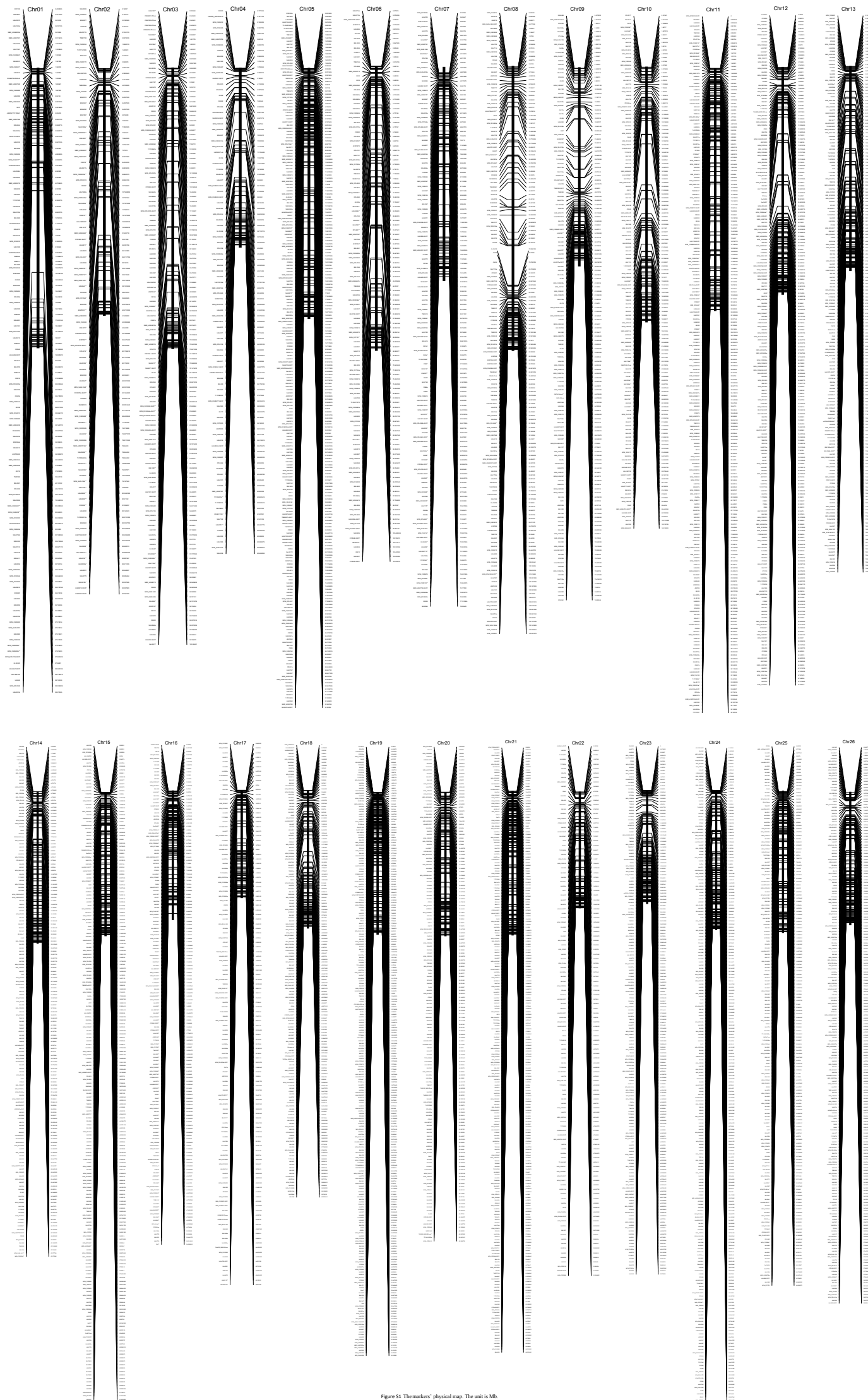

Figure S1 The markers' physical map. The unit is Mb.

Supplement: S1 Fig — (PDF) [file pone.0188682.s001.pdf]
